# Innovative electrode and chip designs for transendothelial electrical resistance measurements in organs-on-chips

**DOI:** 10.1039/d3lc00901g

**Published:** 2024-01-02

**Authors:** Muriel A. Holzreuter, Loes I. Segerink

**Affiliations:** a BIOS Lab on a Chip group, MESA+ Institute for Nanotechnology University of Twente The Netherlands m.a.holzreuter@utwente.nl

## Abstract

Many different epithelial and endothelial barriers in the human body ensure the proper functioning of our organs by controlling which substances can pass from one side to another. In recent years, organs-on-chips (OoC) have become a popular tool to study such barriers *in vitro*. To assess the proper functioning of these barriers, we can measure the transendothelial electrical resistance (TEER) which indicates how easily ions can cross the cell layer when a current is applied between electrodes on either side. TEER measurements are a convenient method to quantify the barrier properties since it is a non-invasive and label-free technique. Direct integration of electrodes for TEER measurements into OoC allows for continuous monitoring of the barrier, and fixed integration of the electrodes improves the reproducibility of the measurements. In this review, we will give an overview of different electrode and channel designs that have been used to measure the TEER in OoC. After giving some insight into why biological barriers are an important field of study, we will explain the theory and practice behind measuring the TEER in *in vitro* systems. Next, this review gives an overview of the state of the art in the field of integrated electrodes for TEER measurements in OoC, with a special focus on alternative chip and electrode designs. Finally, we outline some of the remaining challenges and provide some suggestions on how to overcome these challenges.

## Abstract 2

This review summarizes innovative chip and electrode designs that use transendothelial electrical resistance (TEER) measurements to assess biological barrier properties (figure created with Adobe Firefly).

## Graphical abstract

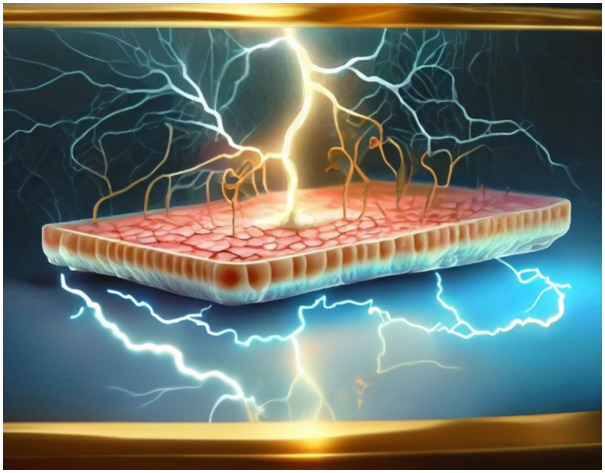

## A Introduction

The development of *in vitro* models that capture the essential aspects of human tissues and organs constitutes an important pillar of modern research into human physiology, disease, and treatments. For many years, static two-dimensional (2D) cultures of different cell types of human or animal origin were the basis of *in vitro* investigations into biological questions. However, in recent years it has become clear that this non-physiological environment may affect cell proliferation,^1^ gene expression,^2,3^ and response to treatments.^4^ In the human body, many cues such as shear stress,^5^ stiffness,^6^ chemical gradients,^7^ and cell contacts^8^ are presented to the cells which are absent in static 2D cultures.

Organs-on-chips (OoC) have become a popular tool to increase the physiological relevance of cell culture models. They allow precise control over different culture parameters, such as through the application of shear stress,^9^ and the creation of chemical gradients^10^ which were challenging in traditional multi-well plates. Additionally, human cells can be incorporated into these models, thus eliminating the issue of interspecies differences. This will also advance personalized medicine by allowing the incorporation of patient-specific cells in the OoC.^11^

However, as models evolve, there is a need for the development of readout methods that are suited to these new models.^12^ In particular, there is an interest in sensors that can provide non-invasive measurements over longer periods of time such that temporal developments can be studied.^13,14^ Fuchs *et al.*^15^ summarized the currently available sensing modalities for on-chip sensing.

One property that is frequently used to monitor *in vitro* models of biological barriers such as the blood–brain barrier (BBB), the gut epithelium, or the pulmonary epithelium, is the permeability of those barriers to different substances. The integrity of these selectively permeable barriers is integral for their physiological functioning since it controls the transport of substances across them. The properties of these barriers can be altered in diseases. For example, disruption of the BBB has been correlated to many diseases such as Parkinson's disease, Alzheimer's disease, and seizures.^16^ Similarly, malfunctioning of the gut barrier has been correlated with inflammatory bowel disease (IBD).^17^ Especially in the context of drug delivery, insights into the transport of therapeutics from the blood across the endothelial barrier into the target tissue are essential.^18^

The integrity of these barrier tissues can be assessed using different tools. Labelled tracers of different molecular weight can be injected on one side of the barrier, and diffusion of the substance over the tissue can be monitored. From the amount of tracer that has crossed the barrier the so-called endothelial permeability coefficient can be calculated. The advantage of this method lies in the selectivity of the measurement. Differently sized compounds may have varying permeability due to the pore size between the tight junctions connecting the cells.^19^ However, oftentimes these measurements can only be performed once on a given cell culture, thus prohibiting dynamic analysis.^20^ Additionally, the coupling of the molecule of interest to a label may interfere with its transport.^21^

Another tool that is often used to assess the barrier integrity is immunofluorescence staining of various proteins involved in the barrier formation such as the tight junction proteins ZO-1 and ZO-2.^20,22,23^ Again, this generally generates only a single time-point and requires a time-intensive process. Furthermore, it is difficult to quantify the results obtained with staining. Although this can be partly solved by performing Western blots,^24,25^ it is still a labour intensive endpoint measurement.

A tool that can be used continuously to measure the barrier integrity utilizes electrical measurements. Electrical impedance spectroscopy (EIS) is a popular label-free approach to investigate the maturity and function of epithelial and endothelial barriers. The opposition of the cell barrier to electrical current flow is related to the tightness and number of intercellular junctions.^26,27^ When cells are connected by tight junctions, the passage of molecules across the barrier is restricted.^28^ This is reflected in an increase of the so-called transendothelial (/-epithelial) electrical resistance (TEER). Many factors such as shear stress,^29^ mechanical strain,^30^ and interaction with other cell types^31,32^ influence the TEER. Further, the shape of the cells forming the barrier can change the measured TEER. A barrier consisting of smaller cells will exhibit a longer cell–cell contact length allowing for more parallel paracellular pathways which will result in a lower TEER.^33^

**Fig. 1:**
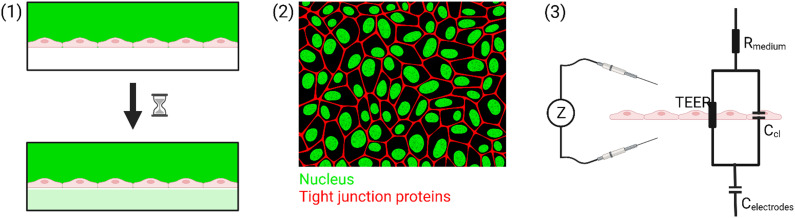
Overview of the different tools commonly used to assess the permeability of biological barriers. (1) Perfusion assay, (2) immunostaining, and (3) TEER. Figure created with https://BioRender.com.

To summarize, there are three common tools that are used to measure the barrier integrity: (1) permeability, (2) (immuno)staining, and (3) TEER. Only the electrical measurements can be done in real-time at multiple time points. In this review, we focus on the technological considerations that need to be taken to make TEER measurements in OoC possible. An overview of the different methods is shown in Fig. 1.

## B Theory of impedance spectroscopy for TEER measurement

Two main approaches are used to determine the TEER [Ω cm^2^] of barrier cultures *in vitro*: the Ohm's law method and impedance spectroscopy. Both of them rely on the same principle: a current signal is applied across the barrier, and the resulting voltage drop is measured.

In the Ohm's law method, the current is applied at a single alternating current (AC) frequency, often at near direct current. Both the current *I* [A] and the voltage *U* [V] are measured across the membrane and are then used to determine the resistance *R* [Ω] using Ohm's law:
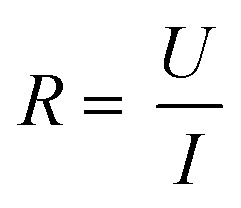
To calculate the TEER, typically the resistance of a blank *R*_blank_ [Ω] is subtracted from the measurement including cells to remove the contribution of the device to the resistance. The resistance is inversely proportional to the culture area, since a larger area creates more parallel pathways for the current to pass through the membrane thus reducing the resistance. Thus, to normalize the measured values, and to make them more comparable between different chip designs, the obtained resistance value is multiplied by the cell culture area *A*_membrane_ [cm^2^]:TEER = (*R* − *R*_blank_)·*A*_membrane_The advantage of Ohm's law method is that the measurements require only simple equipment and are quick since only a single frequency needs to be assessed. Additionally, no data fitting is required to obtain the TEER from the measurements. However, the Ohm's law method fails to collect information contained in the frequency spectrum such as the cell layer capacitance.

Several commercial systems for TEER measurement rely on the Ohm's law method. The EVOM2 (World Precision Instruments) uses an AC square-wave current with an amplitude of 10 μA at 12.5 Hz to measure the resistance at a resolution of 1 Ω. The basic system works with manually placed chopstick electrodes which deliver a non-uniform electrical current density. However, the EVOM2 is compatible with EndOhm Chambers (World Precision Instruments) which allow measurements with higher reproducibility by using fixed circular electrodes instead of the chopstick electrodes. This creates a more uniform electric field and removes errors caused by electrode placement. Another system using chopstick electrodes is the Millicell ERS (Merck Millipore) which uses the same 10 μA amplitude AC square-wave current at 12.5 Hz with a measurement resolution of 1 Ω.

The other approach of obtaining the TEER is impedance spectroscopy (IS). It is a more elaborate technique but it does not assume direct current and measures the capacitance of the cell layer in addition to the TEER which provides more information about the cells.^18^ The capacitance can be related to the morphology of the cells, and can thus be used as a measure for the differentiation of certain cell types such as for the formation of villi in gut epithelial cells.^34^ In IS, an AC current of small amplitude is injected across the barrier, and the resulting voltage drop as well as the phase shift between voltage and current are recorded. This process is repeated for a range of different frequencies, typically from 10 Hz to 100 kHz^35,36^ but sometimes up to 1 MHz,^37^ to obtain an impedance spectrum. The complex electrical impedance *Z* is the AC-analogue of the resistance, and is defined as the opposition to AC flow. It can be calculated from the ratio of the voltage drop Δ*U* and the flowing current *I*:
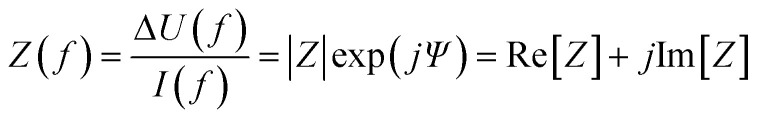
The impedance is described by its magnitude |*Z*| and the phase shift *Ψ* between the voltage and the current. Alternatively, the impedance can be described as the sum of its real and imaginary parts, where the real part of the impedance Re[*Z*] corresponds to the resistive component (frequency independent) and the imaginary part Im[*Z*] corresponds to the capacitive and inductive components (frequency dependent). For the purpose of TEER determination, the focus lies on resistive elements as well as capacitive elements. The combination of these elements leads to three distinct regions in the impedance spectrum in which different parts of the system dominate the impedance.

The TEER is obtained from the measured impedance spectrum in two steps. Firstly, the measured system is described by a so-called equivalent circuit which represents the cell layer and other components contributing to the impedance in an abstracted way. Secondly, the impedance spectrum is fitted to the equivalent circuit which gives a value for the TEER.

A simplified equivalent circuit describing the cell layer generally consists of a paracellular and a transcellular resistance (*R*_para_ and *R*_trans_), as well as a membrane capacitance *C*_cl_ in parallel (see Fig. 2.1).^38^ Additionally, a medium resistance *R*_medium_ and double layer capacitance *C*_dl_ showing the effect of the electrodes on the measurement are added in series. Instead of a double layer capacitance, the electrode-medium interface impedance is better described by a constant phase element, which is a non-ideal capacitor.^39^

**Fig. 2:**
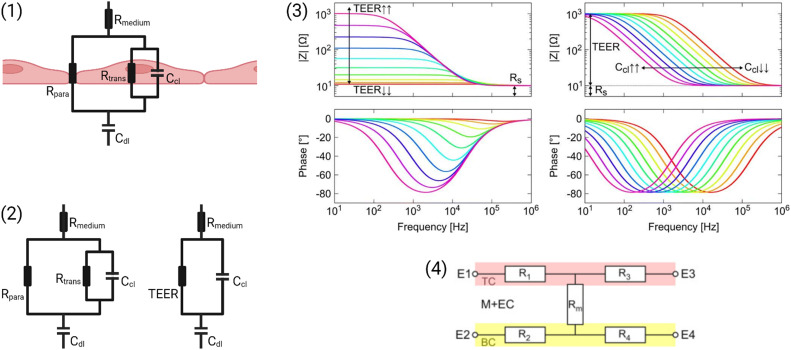
Modelling the cell layer with an equivalent circuit. (1) Cell layer with equivalent circuit. Figure created with https://Biorender.com. (2) Complete *vs.* simplified equivalent circuit. Figure created with https://Biorender.com. (3) Typical impedance spectrum with the impedance (top) and phase shift (bottom). The left impedance spectrum shows the changes in response to different TEER values. The right impedance spectrum shows the changes in response to different cell layer capacitance values. (adapted from ref. 37, copyright 2018 Springer Nature, reproduced *via* Creative Commons Attribution license 4.0 (https://creativecommmons.org/licenses/by/4.0/)). (4) Simplified equivalent circuit of the chip used by van der Helm *et al.*^42^ in order to reduce the effects of confounding factors on the measurements. Reprinted from ref. 42, Copyright (2016), with permission from Elsevier.

Often, this circuit is further simplified (see Fig. 2.2) by neglecting the transcellular resistance *R*_trans_ which is generally much higher and thus does not significantly contribute to the TEER:^18^
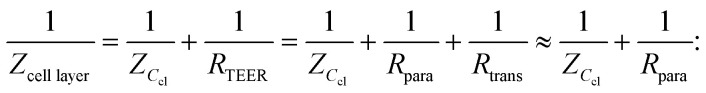
However, in very tight endothelial layers such as the BBB, the paracellular resistance *R*_para_ can become high enough to be in a similar order of magnitude as the transcellular resistance, therefore no longer allowing this simplification.^40^

The impedance measurements can be performed with a two- or four-electrode setup. If two electrodes are used, these are used to both inject the current and measure the resulting voltage drop. However, these measurements will be affected by charge accumulation at the electrodes which contributes to the impedance.^37^ Four-point measurements avoid this disturbance to a large extent by using separate electrode pairs to inject the current (current carrying electrodes, CC) and to measure the voltage drop (pick-up electrodes, PU). Nevertheless, four-point measurements may be influenced by other sources of error.^41^

A commercial system that uses IS for TEER determination is the CellZScope (nanoAnalytics) which measures a frequency range from 1 Hz to 100 kHz, and can measure up to 96 wells automatically depending on the model. Another commercial impedance spectroscope used for TEER detection is the Locsense Artemis (Locsense). The instrument can be coupled to both transwell plates as well as microfluidic chips, and measures in a frequency range from 10 Hz to 100 kHz.

There are many factors that influence the measured TEER. Most importantly, the impedance changes as cells grow, proliferate, and form a tighter barrier. The effect of those changes on the impedance spectrum are shown in Fig. 2.3. However, apart from these factors of interest, the impedance is also influenced by temperature or composition of the medium, air bubbles in the channels, or positioning of the electrodes.^42^ The following sections will examine these influences in more detail.

Firstly, Odijk *et al.*^43^ have shown that one missing cell in the barrier monolayer already decreases the measured TEER significantly. A drop in cell coverage from 100% to 99.6% causes an 80% decrease in the measured TEER. This highlights the importance of achieving a confluent monolayer to accurately determine the TEER. Additionally, when cells are cultured on a porous membrane, tight adhesion of the cells to the membrane may already increase the measured impedance.^44^

Next, the applied transmembrane potential to measure TEER must be kept significantly below 250–350 mV to avoid membrane electroporation.^45^ The applied currents may also influence cell behaviour at lower amplitudes. Typically used current amplitudes for TEER measurements are in the order of tens of μA.^34,46^

Additionally, the right choice of materials for the electrodes is of importance. Platinum^35,47,48^ and gold^49–51^ are some of the most commonly used electrode materials in OoC. They have the advantage of being inert and biocompatible.^15^ The downside of these materials is that they can suffer from a high electrode-electrolyte interface impedance due to their polarizability which can cover up small changes in the TEER.^15,52^ Electrodes are also frequently made of Ag/AgCl^32,53,54^ which has a lower interface impedance due to its non-polarizability.^15,52^ However, it has been shown that silver ions can have cytotoxic effects^55,56^ which is why silver electrodes may not be compatible with long-term monitoring applications. Another material that has been used for electrodes is indium tin oxide (ITO)^57–59^ due to its transparency and high electric conductivity.

Furthermore, it is important to keep in mind that the low channel volumes of microfluidic chips have a high resistance compared to the expected TEER values of the barriers. The contribution of the medium in the channel to the overall measured resistance can be approximated with the equation:
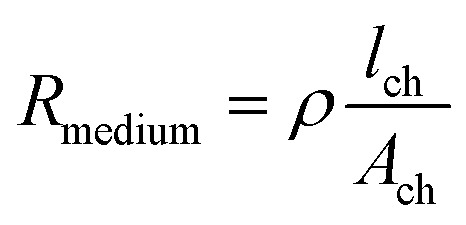
where *R*_medium_ is the medium resistance, *ρ* is the specific resistance of the medium, *l*_ch_ is the length of the channel, and *A*_ch_ is the cross-sectional area of the channel.^42^ This formula demonstrates that medium contribution to the measured impedance is particularly influential for long channels with a small cross-sectional area. This may cause problems since variations in the medium resistivity due to temperature fluctuations overshadow the changes in TEER due to barrier maturation or drug perturbation.^43^

Van der Helm *et al.*^42^ found a method to exclude the effects of changes to non-biological factors such as temperature or electrode position from the measured TEER. This is done through six measurements between four electrodes. The measured resistances between the different electrode pairs can be described using five unknown partial resistances (see Fig. 2.4) which can be determined by Gaussian elimination. This way, the resistance of the membrane and cell layer (*R*_m_) can be determined isolated from the resistance contributions of the channels.^42^ IS allows the determination of the medium resistance from the impedance spectrum instead of from a blank control. In this manner, it serves as an intrinsic temperature control since the obtained medium resistance varies with a change in temperature of the chip.^26^

Finally, the shape and relative position of the electrodes can influence the measurements through different current distributions across the culture area. Because of this, different areas of the barrier may not contribute equally to the measured impedance. This effect is quantified by the impedance sensitivity *S* which indicates the contribution of an area to the total measured impedance:^41^
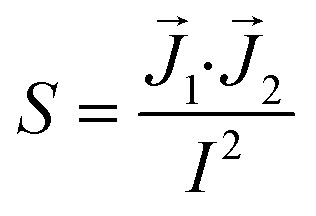
where the vector *J⃑*_1_ is the current density when injecting a current *I* between the CC electrodes, and *J⃑*_2_ is the current density when injecting a current *I* between the PU electrodes. If the sensitivity of an area is positive, an increase in the local impedance will cause an increase in the measured impedance. The absolute value of the sensitivity indicates the magnitude of the influence on the measured impedance.^41^ It is important to note that the impedance sensitivity is dependent on the TEER. Generally, the sensitivity is distributed more evenly across the barrier for a higher TEER.^37^ Yeste *et al.*^60^ proposed an interdigitated electrode design to optimize the sensitivity while allowing visual inspection of the cultured cells.

To facilitate comparison between different chip designs, the effect of unequal barrier contributions of different areas can be compensated by multiplying the measured TEER by a geometric correction factor (GCF) which was proposed by Yeste *et al.*^37^ The GCF takes chamber geometry and electrode placement into account, and is calculated as the ratio between the theoretical TEER_t_ used as an input for finite element modelling, and the simulated measured TEER_s_:
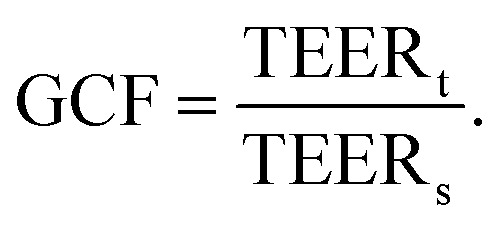
The corrected TEER_GCF_ can then be calculated using the formulaTEER_GCF_ = (*R* − *R*_blank_)·*A*·GCF.

## C State of the art

Electrodes for the determination of TEER have been integrated into many different OoC already. In this section, we will give an overview of the different chip and electrode designs that have been explored so far. The first part will focus on the conventional sandwich chip design that consists of a top and a bottom channel separated by a permeable membrane. In the following parts, alternative designs will be discussed. All designs are summarized in Table 1.

**Table 1:** Summary of different electrode designs and the OoC they have been used for

Organ	Cell types	Chip type	Materials	Channel dimensions	Electrodes	Current frequency range	Flow	TEER (careful, units are not always the same!)	Source
BBB	Human hippocampal astrocytes, hCMEC/D3, human brain vascular pericytes	Parallel channels, fibrin hydrogel	PDMS, glass	Vascular channel *w* = 300 μm	Gold, circular, *d* = 150 μm, 150 μm apart	10 Hz–1 MHz	No	12 480 Ω cm^2^	Palma-Florez *et al.*^49^
	hCMEC/D3, normal human astrocytes	Sandwich-chip	PDMS, PET membrane, glass	Cell culture area: *d* = 4 mm, *h* = 1 mm	Gold, rectangle	1 Hz–100 kHz	No	800–3400 Ω cm^2^	Yang *et al.*^64^
	hiPSC-derived cells: hiBMEC, hiAstro	Sandwich-chip	OSTE+, PC membrane	*h* = 250 μm, 200 × 15 mm^2^	Gold, interdigitated	EVOM2 (12.5 Hz)	Yes (peristaltic pump, 1.5 μL min^−1^)	490 Ω	Matthiesen *et al.*^51^
	Human astrocytes, HUVECs, human brain vein pericytes (HBVP)	Sandwich-chip	PDMS, Whatman qualitative filter paper: grade 2	*l*/*w*/*h* = 30 mm, 2 mm, 1 mm	Aluminium sheet (cleanroom-free)	EVOM2 (12.5 Hz)	No	330 Ω cm^2^	Duong *et al.*^62^
	Primary neonatal rat brain EC, pericytes and astrocytes	Viscous-finger patterned channel	PDMS, collagen I gel	Hollow channel diameter = 300–400 μm	One in media reservoir, one fixed to inner surface of PDMS channel (outside EC layer)	EVOM2 (12.5 Hz)	Yes (gravity driven flow with a paper flow resistor, 50–100 μL h^−1^)	—	Yu *et al.*^67^
	Human brain microvascular endothelial cells (hBMVEC)	Single channel	PDMS, well from an 8-well plate, glass	*w* = 400 um, *l* = 2.9 mm	Gold, array of circles along the channel	10 Hz–1 MHz	No	—	Young *et al.*^50^
	hCMEC/D3	Sandwich-chip	PDMS, PC membrane	*l*/*w*/*h* = 1 cm/500 μm/100 μm	Platinum wire (*d* = 200 μm)	100 Hz–10 MHz	Yes (2.5 mL h^−1^)	36.9 Ω cm^2^ (day 3)	Griep *et al.*^47^
	b.End3 endothelial cells, C8D1A (astrocytes)	Sandwich-chip	PDMS, PC membrane	*l*/*w*/*h* = ?/(2 mm, 5 mm)/200 μm	AgCl thin-film (75% of cell culture area)	EVOM2 (12.5 Hz)	Yes (peristaltic pump, 1.3–2.6 μL min^−1^)	>250 Ω cm^2^	Booth and Kim^32^
	Primary mouse astrocytes, primary mouse BMEC	Sandwich-chip	PDMS, PC membrane	*l*/*w*/*h* = 19 mm, 1 mm, 300 μm	Gold on PC substrate	EVOM2 (12.5 Hz)	Yes (syringe pump, 1.5 μL min^−1^)	4.48 ± 0.79 kΩ (co-culture)	Jeong *et al.*^71^
Gut	Caco-2	Sandwich-chip with a circular insert	COP, PET membrane	Upper: 25 mm × 2.6 mm × 1 mm lower: 30.3 mm × 2.6 mm × 1 mm	PEDOT:PSS, *d* = 6.5 mm (whole cell culture area)	10 Hz–100 kHz	Yes (peristaltic pump, 20 μL min^−1^)	1000 Ω cm^2^	Marrero *et al.*^69^
	Caco-2	Sandwich-chip	PDMS, PC membrane	Upper: 18 mm × 1 mm × 1 mm lower: 18 mm × 1 mm × 0.5 mm	Bottom: gold (2 mm wide), top: stainless steel wires (movable)	1 Hz–1 MHz	No	—	Renous *et al.*^63^
	Caco-2	Sandwich-chip	TOPAS polymer platform, PET membrane	5 mm × 7.3 mm × ?	ITO, covering entire culture area	100 Hz–10 kHz	Yes (7 μL min^−1^)	1000 Ω cm^2^	Giampetruzzi *et al.*^57^
	Caco-2	Parallel channels	Organoplate, ECM hydrogel	—	Stainless steel chopstick	0.1 Hz–1 MHz	Yes (rocking plate)	588 Ω cm^2^	Nicolas *et al.*^73^
	HT29 human colon adenocarcinoma cells, MDCK cells	Parallel channels	COC, Cellendes 3-D Life hydrogel	*h* = 170 μm	Gold	1 Hz–10 kHz	Yes	50–80 Ω cm^2^	Nair *et al.*^65^
	Caco-2	Sandwich-chip	PDMS, PDMS membrane	Upper: 24 mm × 1 mm × 1 mm lower: 30 mm × 1 mm × 0.2 mm	Platinum wire (cleanroom-free)	100 Hz–1 MHz	No	9.3–13 kΩ (day 21)	Bossink *et al.*^61^
	Caco-2	Sandwich-chip	PDMS, PC, PDMS membrane	Upper: *h* = 1 mm lower: *h* = 0.2 mm	Gold (semi-transparent), *w* = 1 mm, sep = 1 mm	100 Hz–10 kHz	Yes (syringe pump, 30 μL h^−1^)	736 ± 120 Ω cm^2^ (one week)	van der Helm *et al.*^34^
	Caco-2	Sandwich-chip with porous villi insert instead of membrane	3D printed PEVA	10 mm × 10 mm	Silver wire	EVOM2 (12.5 Hz)	Yes	—	Costello *et al.*^53^
	Colon cells	Sandwich-chip (multiplexed)	COP, track-etched membrane	*w* = 1 mm, *h* = 0.25 mm	Chopstick in inlets and outlets	—	Yes (pneumatic actuation, 48–600 μL min^−1^)	234.5 ± 33.7 Ω cm^2^	Azizgolshani *et al.*^72^
Lung	Human non-small cell lung cancer cell line A549	Sandwich-chip	PDMS, PET membrane	*l*/*w*/*h* = 10 mm/1 mm/10 μm	ITO, covering entire culture area	1 Hz–1 MHz	No	—	Liu *et al.*^70^
	Primary human airway epithelial cells	Sandwich-chip	PDMS, PET membrane	*h* = 1 mm/0.2 mm	Gold on PC substrate, 1 mm wide	10 Hz–100 kHz	Yes (60 μL h^−1^)	51 Ω cm^2^ (day 62)	Henry *et al.*^36^
Skin	Primary human foreskin-derived dermal fibroblasts (HDFn), primary human epidermal keratinocytes	Sandwich-chip	PDMS, polystyrene		0.8 mm diameter Ag/AgCl wire	12.5 Hz	Yes (1.5 μL min^−1^)	1050 Ω cm^2^ (day 12)	Zoio *et al.*^54^
Heart	HUVEC, hiPSC-derived cardiomyocytes	Sandwich-chip	PDMS, PET membrane	Upper: 2 cm × 1 mm × 0.4 mm lower: 2 cm × 1 mm × 1 mm	Platinum on PC (top) or glass (bottom), 1400 μm × 500 μm	10 Hz–100 kHz	Yes (peristaltic pump, 60 μL h^−1^)	232 ± 47 Ω	Maoz *et al.*^35^
Blood vessel	HUVEC	Circular channel in hydrogel	Collagen gel in 3D printed chip	*d* = 500 μm	Ag wire coated with parylene, *d* = 100 μm	Millicell ERS-2	Yes (peristaltic pump)	60 Ω cm^2^ (day 2)	Mori *et al.*^66^
Retina	ARPE-19 cells, primary HREC, human neural cells (SHSY5Y cell line)	Parallel channels connected by microgrooves	PDMS, glass	Channels: *w* = 500 μm, *h* = 230 μm microgroves: *w* = 2 μm, *h* = 4 μm	Platinum black, 40 μm × 4 mm	100 Hz–1 MHz	Yes (syringe pump, 0.1 mL min^−1^)	ARPE-19: 3.2 ± 0.46 kΩ HREC: 2.9 ± 0.73 kΩ (day 3)	Yeste *et al.*^48^

### Different electrode designs in conventional sandwich chips

Already the very first BBB-on-chip presented by Booth and Kim^32^ included electrodes for the determination of TEER. In their chip design, the top and bottom channel run perpendicular to each other. At their cross-section, a porous polycarbonate membrane separates the channels and allows for limited interaction between the cells cultured on either side. Silver electrodes were patterned on the top and bottom glass slide using sputter deposition. Cells were continuously perfused using a peristaltic pump with a flow of 2.6 μL min^−1^. Using this setup, cell layers consistently reached TEER values above 250 Ω cm^2^, significantly higher than the transwell controls with TEER values of 25 Ω cm^2^. TEER values additionally increased in co-culture with astrocytes. However, due to the very small pore size (0.4 μm), direct contact between the cell types was unlikely.

Henry *et al.*^36^ used e-beam evaporation to integrate semi-transparent gold electrodes (1 mm wide, spaced 1 mm apart) into their polycarbonate (PC) chips to allow for the visual inspection of cells inside the channels (see Fig. 3.1). Inside the bottom channel, human airway epithelial cells (hAECs) were cultured for six days with media in both channels before the creation of an air–liquid interface (ALI). Cell culture was continued for a total of 62 days. TEER was determined through 4-point impedance measurements covering a frequency range of 10 Hz to 100 kHz. The observed resistance plateaued at an average of 1700 Ω after the establishment of ALI.

**Fig. 3:**
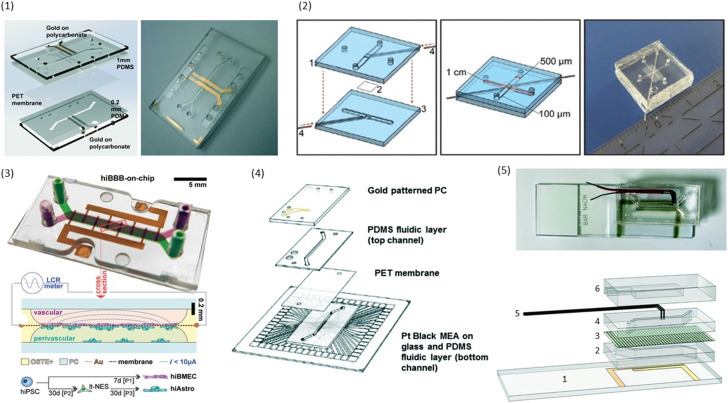
Examples of conventional sandwich chips enabling TEER measurements. (1) CAD model and photograph of the TEER-chip designed by Henry *et al.*^36^ Reproduced from ref. 36 with permission from the Royal Society of Chemistry. (2) Exploded schematic and photograph of the BBB-on-chip by Griep *et al.*^47^ with Pt wires for electrodes. Reproduced with permission from ref. 47, copyright 2012 Springer Nature. (3) Top: photograph of the BBB-on-chip by Matthiesen *et al.*^51^ The electrodes are integrated onto the membrane instead of above and below the channel. Bottom: schematic of the channel cross-section with current flow indication. Reproduced with permission from ref. 51. Copyright 2021 The Authors. Published by Wiley-VCH GmbH. (4) Exploded schematic of the TEER-MEA chip from Maoz *et al.*^35^ The chip includes electrodes above and below the membrane. Additionally, the bottom channel contains a MEA to measure the activity of electrically active cells. Reproduced from ref. 35 with permission from the Royal Society of Chemistry. (5) Photograph (top) and exploded view (bottom) of the spatial-TEER (sTEER) device developed by Renous *et al.*^63^ The device features bottom electrodes that are fixed in position, as well as top electrodes that can be moved along the microfluidic channel to determine the TEER in different areas of the channel. In the exploded view, the assembly of the chip with its components is show: 1 – glass cover slip with patterned gold electrodes, 2 – bottom PDMS channel, 3 – permeable PC membrane, 4 – top PDMS channel, 5 – movable stainless steel electrodes, 6 – top PDMS layer. Reproduced and adapted from ref. 63 with permission from the Royal Society of Chemistry.

To allow cleanroom-free integration of cheap electrodes, Griep *et al.*^47^ utilized inert platinum (Pt) wires as electrodes in a PDMS chip with a porous polycarbonate membrane. The wires were placed in dedicated grooves in the PDMS and fixed with an optical adhesive to prevent movement (see Fig. 3.2). A BBB-on-chip was established using the immortalized human brain endothelial cell line hCMEC/D3. Staining for ZO-1 confirmed the formation of tight junctions in the monolayer. The measured TEER reached a plateau of 36.9 Ω cm^2^, similar to the values obtained in transwell monocultures of hCMEC/D3. After the application of 5.8 dyn cm^−2^ (= 5.8 kPa m^−2^) of shear stress for 18 hours, the measured TEER tripled to reach up to 120 Ω cm^2^. Follow-up research of the same group used a similar strategy to integrate electrodes into chips with a larger intersection between the top and the bottom channel.^61^

An alternative way of integrating cheap electrodes without the use of cleanroom facilities was explored by Duong *et al.*^62^ The group cut aluminium sheets to size, and integrated them directly into a PDMS microfluidic chip with a cellulose fibre membrane. They then cultivated HUVECs, human astrocytes (HA) and human brain vein pericytes (HBVPs) inside the chip to form an *in vitro* BBB. Over a culture period of 7 days, an increase in TEER up to 330 ± 4.16 Ω cm^2^ was observed.

Instead of measuring TEER, Matthiesen *et al.*^51^ utilized electric cell–substrate impedance sensing (ECIS) to assess the barrier properties in a neurovascular unit (NVU)-on-chip. They patterned interdigitated gold electrodes on the polycarbonate membrane used to separate the top and bottom channel of the microfluidic chip made from off-stoichiometry thiol–ene–epoxy (OSTE+) as can be seen in Fig. 3.3. By incorporating the membrane inside the OSTE+ during the chip moulding, they could reduce the amount of alignment and assembly steps needed. Their fabrication process additionally allowed the fabrication of rounded corners touching the membrane which improves cell adherence at the edges of the channels. A single read-out frequency of 6 kHz was used to determine the ECIS. They could show continuous tight junction formation between the endothelial cells, and demonstrated the high temporal resolution of barrier integrity measurements using ECIS by perturbing the BBB-on-chip with reactive oxygen/nitrogen species (RONS), and monitoring the drop in TEER. However, ECIS assesses TEER only locally, and thus should be combined with a more global technique such as tracer dye permeability.^51^

In addition to assessing TEER inside their chip, Maoz *et al.*^35^ also integrated a multielectrode array (MEA) into the bottom channel of their design (see Fig. 3.4). This allowed them to measure the electrical activity of human induced pluripotent stem cell-derived (iPSC) cardiomyocytes, while also measuring the barrier integrity of the endothelial layer in the top channel. With this system, they were able to show differences in the response of cardiomyocytes to the β1-adrenergic agonist Isoproterenol depending on whether the drug was administered directly into the cardiomyocyte compartment, or whether it was administered over an intact or inflamed endothelium.

In an attempt to obtain more spatial information about local TEER values inside a chip, Renous *et al.*^63^ designed a device with movable electrodes for TEER determination (see Fig. 3.5). The position of the two top electrodes could be controlled with a precision of ±10 μm, whereas the two electrodes in the bottom compartment were in a fixed position spanning the entire length of the channel to avoid variations in electrode separation. The use of transparent gold bottom electrodes allows for visualization of the cells inside the channel. TEER was then determined using four-point measurements. Using Caco-2 cell cultures, they were able to show that spatial variations of TEER could be detected with this system.

The chip design of Young *et al.*^50^ also provides insight into spatial distribution of TEER. In this chip, an array of electrodes was patterned on the bottom slide of the channel. By measuring the impedance between nearest neighbouring electrode pairs, a map of impedance values along the channel was created. Additionally, electrodes only covered a small portion of the channel which allowed for visual inspection of the cells.

### Alternative chip and channel designs

The chip designs mentioned so far were based on the basic structure of having a top and bottom channel separated by a permeable membrane. However, this chip design does not recapitulate the aspects of the native environment of the cells.

In an attempt to more closely model the native structure of the small intestine, Costello *et al.*^53^ designed a porous villi scaffold made from polyethylene–vinyl–acetate (PEVA) that fits into a perfusable bioreactor, creating an apical and basal compartment. Silver wires in the apical and basal compartment served as electrodes for TEER measurements. Using this design, they could monitor the TEER of Caco-2 cell layers under static or flow conditions. They were also able to show that the differentiation and proliferation profile of cells grown on the villi scaffold more closely mimics the *in vivo* situation.^53^ However, silver chloride electrodes have been shown to have a cytotoxic effect when exposed to the cell culture medium over an extended period of time.^45^

Similarly, Zoio *et al.*^54^ designed a modular chip that also allows the measurement of TEER across thicker cell constructs cultured in a 3D scaffold instead of on a membrane. The chip fabrication required no cleanroom access or plasma-bonding steps, and the chip could be disassembled to remove the cultured tissue for further analysis. TEER measurements were conducted in real-time with integrated electrodes. COMSOL simulations demonstrated a more evenly distributed sensitivity across the cell culture area with a maximum sensitivity deviation of 10% for the integrated electrodes *versus* a maximum deviation of 17% for chopstick electrodes. Additionally, *in situ* measurements showed a lower measurement variability (5% maximum deviation) for the integrated electrodes. They validated the designed chip by culturing a full-thickness skin model (FTSm) on the scaffold with an air–liquid interface. The FTSm showed good maturation and reached TEER values of 1050 ± 180 Ω cm^2^, comparable to previously published literature. Further, a decrease in TEER could be observed in real-time in response to a benchmark irritant.

Another chip design which can be assembled after cell seeding was developed by Yang *et al.*^64^ To ensure a more uniform cell seeding density, the microfluidic channels were only closed off after cells had been seeded on either side of the porous membrane. The chip was then assembled by sandwiching the PDMS channel structures with the integrated membrane in between two glass slides with patterned electrodes for 4-point electrical impedance measurements. No additional bonding steps were required for the assembly which allows for the reusing of the electrodes. They observed a reduction in cell loss during seeding for the open channel chip compared to the same design with seeding through the inlets. However, this setup requires many manual assembly steps which may reduce the repeatability of the performed experiments. Additionally, since the different chip components are only pressed together, and not bonded to each other, leaking may become an issue.

To avoid the use of artificial membranes separating the channels, Palma-Florez *et al.*^49^ develop a different chip design that allows for the determination of TEER in a chip with a hydrogel-filled central channel. Using photolithography, they patterned gold electrodes on a bottom glass slide which was then bonded to a PDMS chip containing three parallel channels (see Fig. 4.1). The central channel was filled with a fibrin hydrogel containing human astrocytes and pericytes, and one of the lateral channels was seeded with hCMEC/D3 in order to mimic the BBB. The electrodes at the bottom of the channels were located close to the hydrogel boundary and allowed for TEER measurements across the endothelial cell layer forming on that boundary. The small separation distance of the electrodes reduces the influence of factors other than barrier integrity on the measured impedance. Additionally, multiple electrode pairs were patterned on the chip, allowing for TEER measurements in different locations. The chip design was then used to evaluate the toxicity of gold nanorods intended for the treatment of Alzheimer's disease.

**Fig. 4:**
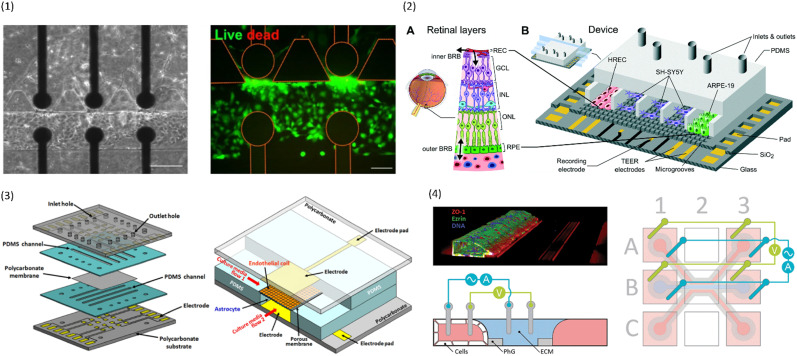
Examples of multiplexed chips for TEER measurements. (1) BBB-on-chip by Palma-Florez *et al.*^49^ Left: brightfield microscopy of the hydrogel zone as well as the endothelial channel. Right: Live-dead staining of the cells in the endothelial channel. Scale bars 100 μm.^49^ Copyright 2023 Springer Nature, reproduced *via* Creative Commons Attribution license 4.0 (https://creativecommons.org/licenses/by/4.0). (2) Model of the blood–retinal barrier by Yeste *et al.*^48^ A: Schematic of the different cell layers of the blood–retinal barrier. B: Schematic of the chip design featuring a network of microgrooves to connect the different cell compartments. Electrodes for TEER measurements are patterned at the bottom of the microgrooves. Reproduced from ref. 48 with permission from the Royal Society of Chemistry. (3) Illustration of the different layers and the assembled chip by Jeong *et al.*^71^ The chip contains four top and four bottom channels that intersect in 16 places. At each intersection, electrodes for TEER measurement are placed above and below the membrane. Reproduced with permission from ref. 71, copyright 2023 IEEE. (4) Confocal image of Caco-2 cells growing inside the commercial OrganoPlate (MIMETAS) as well as schematic of the electrode placement inside the media reservoirs. Reproduced from ref. 73, copyright 2021, with permission from the Royal Society of Chemistry *via* Creative Commons Attribution 3.0 Unported Licence (https://creativecommons.org/licenses/by/3.0/).

A similar chip was designed by Nair *et al.*^65^ Two parallel channels are separated by pillars which keep the hydrogel to one compartment. Gold electrodes were evaporated on the sides of the channels to allow for TEER measurements. They cultured HT29 Human colon adenocarcinoma cells inside the hydrogel, and lined the vessel compartment with MDCK cells. With this design, it could be observed how tumour cells would remodel their environment, and eventually migrate into the vascular compartment for leaky barriers.

Another approach to circumvent the use of artificial membranes was presented by Yeste *et al.*^48^ By creating a grid of microgrooves, they connected parallel channels to each other (see Fig. 4.2). At the bottom of the grid, electrodes were integrated to measure the electric activity of neuronal cells, as well as for TEER measurements. Instead of measuring the TEER by placing electrodes on either side of the membrane, both electrodes were located on the basal side. In this setup, the current will pass across the cell barrier twice. The group then compared the measurements obtained using this setup to the traditional electrode setup, and were able to show that they produce comparable measurements for cell cultures of ARPE-19 during a challenge with EDTA.

To get even closer to the native structure of a vessel, Mori *et al.*^66^ introduced electrodes for TEER measurements into a vessel fully suspended inside a hydrogel. They did so by casting the hydrogel around a syringe needle with silver wires glued to its tip. When extracting the needle, a circular channel with a diameter of 500 μm was created inside the hydrogel, and the electrode wires were inserted simultaneously. The HUVECs seeded into the device were able to grow and proliferate inside the circular channel. Furthermore, the authors showed that HUVECs cultured inside the channel under perfusion maintained their TEER longer compared to HUVECs cultured inside the channel under static conditions.

Another chip design integrating electrodes into a fully suspended hydrogel channel was presented by Yu *et al.*^67^ They created a channel inside a collagen gel using viscous finger patterning. This setup allowed them to integrate astrocytes directly into the hydrogel for co-culture with the endothelial cells. Additionally, pericytes lined the created vessel together with the endothelial cells. TEER was measured using a EVOM2 volt–ohm meter by placing one electrode in the chip inlet, and the other in the microfluidic channel outside the endothelial layer. Using this chip design they could show the establishment of a tight barrier as demonstrated by increasing TEER. Further, the created vessel displayed a drop in TEER in response to the inflammatory agent tumour necrosis factor α (TNF α), as would be expected from *in vivo* studies.

### Dealing with measurement error

While TEER measurements are simple to execute, it is important to keep various different error sources in mind, and optimize chip design as well as data processing in order to make comparison between different chip designs possible. In the theory section of this review, we explained the possibility of using the geometric correction factor to compensate errors arising from different chip designs. In this section, we will present some alternative approaches that have been taken.

A similar approach to the geometric correction factor was used by van der Helm *et al.*^34^ who modelled the chip with a 2D electrical network using four different types of elements. The four elements represented the culture medium, permeable membrane, cell layer, or the double layer capacitance at the electrode–medium interface. The potential of each network node could be calculated using the input current together with Ohm's law and Kirchhoff's law. From the obtained potentials, the sensitivity distribution could be calculated and used to correct the measurements. The group was also able to study the dependence of the measured impedance on the input TEER and the frequency.

Instead of only correcting for the TEER using sensitivity analysis, Miyazaki *et al.*^68^ used finite element analysis to optimize the sensitivity distribution across the cell culture area by adjusting their electrode design. In addition to achieving a uniform sensitivity distribution, they set the goal to allow for cell observation inside the chips. In their final design of thin (10 μm wide) interdigitated electrodes, sensitivity variation is as low as 0.041%, well below the set 5% threshold. Further, thanks to the small electrode width compared to electrode spacing, cells can be observed in the entire culture area because the electrodes are not in the same focal plane.

Marrero *et al.*^69^ tackled the issue of uneven sensitivity distribution by covering the entire cell culture area with electrodes. They determined TEER using a bipolar setup. In order to still be able to observe the growing cell layer, they utilized the semi-transparent organic semiconductor polymer poly(3,4-ethylenedioxythiophene) doped with polystyrene sulfonate (PEDOT:PSS). PEDOT:PSS has the advantage over inorganic semi-transparent electrodes due to its lower electrode polarization impedance. The chip design was validated by characterizing the electrochemical and optical properties of the electrodes, as well as by monitoring the TEER values of Caco-2 cells for seven days. Additionally, the observation of a drop in TEER in response to EDTA treatment demonstrated sufficient time-resolution of the measurement system.

An interesting approach to compensate for measurement errors due to microbubbles in the channels was presented by Giampetruzzi *et al.*^57^ In their chip design, the transparent indium tin oxide (ITO) electrodes cover the entire cell culture area above and below the porous PET membrane. Every impedance measurement was accompanied by a photograph of the culture area. Later on when performing the area correction of the TEER, only the culture area not covered by microbubbles (assessed using ImageJ) was used in the calculation.

Instead of correcting for the effective area through image analysis, Liu *et al.*^70^ presented a new equivalent circuit model in order to obtain more accurate estimates of the TEER. To reduce the influence of gaps in the cell layer on the measured TEER, they estimated the cell coverage using the measured capacitance. Next, they calculated the ratio of the cell layer area to the total culture area. They then used this ratio to determine the influence of gaps in the cell layer on the TEER.

### Multiplexing

While it is important to achieve accurate and comparable measurements from the individual chips, another concern in the field of OoC is throughput. In order to be useful to pharmaceutical companies and large-scale research, many chips must be run in parallel with as little human intervention as possible.

To achieve a high level of multiplexing, Jeong *et al.*^71^ designed a chip with four bottom and four top channels that intersect in a total of 16 places. At each channel intersection, the top and bottom layer are separated by a permeable polycarbonate membrane, and electrodes are placed above and below the channel to allow for TEER measurement (see Fig. 4.3). Primary mouse endothelial cells grew into a continuous monolayer inside the chip, and demonstrated an increased TEER under flow culture conditions. Further, the authors were able to show the protective effects of astrocytes on endothelial cell culture during histamine treatment. While the TEER of endothelial monocultures drastically reduced in response to histamine, the TEER of endothelial cells in coculture with astrocytes remained constant.

Azizgolshani *et al.*^72^ tackled the issue of low throughput by designing a microfluidic culture plate containing 96 sandwich chips in parallel in a traditional well plate format. This ensures compatibility with automated liquid handling and plate shuttling infrastructure to reduce manual labour. The culture plate was equipped with up to 192 microfluidic pumps to individually address the flow of each channel. Additionally, chop stick electrodes were integrated into the culture plate lid, and dip into the inlets and outlets of the chips to allow for TEER measurements across the membrane. While this device design allows for high throughput, the chopstick electrodes lead to a non-uniform current density, particularly at low TEER which can lead to inaccurate representation.

To avoid artificial membranes inside the chip design while increasing throughput, Nicolas *et al.*^73^ used the commercially available OrganoPlate platform (MIMETAS, Leiden, The Netherlands), which contains 40 parallel-channel chips with a hydrogel-filled centre channel (see Fig. 4.4). The channel separation occurred through phaseguides keeping the hydrogel in the centre channel. TEER was determined from electrodes inserted into the medium reservoirs of the different channels by applying a sinusoidal AC voltage at an amplitude of 100 mV with frequencies ranging from 0.1 Hz to 1 MHz. A measurement module was coupled to the electrode board which allowed automatable TEER measurements of the entire plate. One of the outer channels was seeded with either Caco-2 cells or with renal proximal tube cells. The two models produced a TEER of 588 Ω cm^2^ ± Ω cm^2^ and 6.4 Ω cm^2^ ± 0.13 Ω cm^2^, respectively. In response to treatment with staurosporine, a decrease in TEER of the Caco-2 culture was shown. The OrganoPlate was placed on an interval rocker for gravity-driven perfusion. Possibly, different cell types could be integrated in the hydrogel for co-culture systems. However, the contact area between the vessel channels and the hydrogel is relatively small compared to the entire vessel wall. Additionally, the phase-guide created border leads to a non-physiological shape of the vessel channel which may hinder cell adhesion. Further, the current field distribution across the barrier was not characterized.

## D Limitations and future directions

While OoC with integrated sensing modalities to measure TEER have made great advances in the past years, some challenges still need to be addressed.

For one, the most commonly used chip design with integrated electrodes is a sandwich-type chip where a top and bottom channel are separated by a permeable membrane. While this design allows for the application of shear stress, the cells are still cultured on a flat surface, and interaction of cells on the two sides of the membrane is hindered. Some alternative designs were presented in the previous section of this review. However, these chip designs come with other limitations such as an uneven current field distribution. This in turn leads to an uneven sensitivity distribution which may cause inaccurate measurements that are difficult to correct. Therefore, more research is needed into innovative chip designs that allow accurate TEER measurements in a physiologically relevant environment for the cells.

Further, the differences in chip and electrode designs make it challenging to compare results from one study to another. Particularly, many publications lack detailed descriptions of the electrode layout and design, and do not quantify the sensitivity distribution across the cell layer. Correcting the TEER values using the geometric correction factor (GCF) as suggested by Yeste *et al.*^37^ allows for more robust comparison. Alternatively, standardization of designs will improve translatability of findings. Additionally, standardization will increase user acceptance and adaptation since end users will only need to learn how to use one system. If done correctly, it will also facilitate the automatization of processes in the lab using existing infrastructure. This in turn would promote the use of OoC in large-scale pre-clinical trials.

In line with the goal of promoting OoC with integrated electrodes for drug research, multiplexing of the OoC is essential. Currently, most chip designs with integrated electrodes contain a single OoC, thus limiting throughput. In order to be useful for applications in drug development and personalized medicine, increasing throughput by multiplexing the chips is essential. This way, many drugs can be screened in parallel with a higher reliability than for normal 2D cell culture, but with lower complexity and cost than for animal models.

Additionally, fabrication of the chips currently rely on many manual assembly steps as well as expensive fabrication tools. For OoC with integrated electrodes to become more widely used, chip designs must be adapted such that they can be fabricated in large quantities at relatively low cost. In this process, PDMS will most likely be replaced with alternative materials. This will be beneficial due to the drawbacks of PDMS such as the absorption of small molecules. Additionally, exploring new materials such as electrically conductive polymers for the fabrication of the chips and the electrodes may lead to improved designs.

Another issue that requires further investigation is the influence of confounding factors and noise on the measured impedance such as temperature and media composition. Additionally, with the incorporation of hydrogels into the microfluidic chip designs, scientists need to account for the influence of hydrogel degradation or cells growing inside the hydrogel on the measured impedance. Therefore, even though TEER measurements are a great way to monitor the cell cultures in a continuous and non-invasive manner, chip designs should also be compatible with alternative readout methods and sensors such as brightfield and fluorescent microscopy in order to confirm the results obtained from the TEER measurements.

Furthermore, one needs to ensure that the voltages and currents applied are low enough to not affect the cells. In line with the goal of not disturbing the cells, choosing the right material for the electrodes is of essence. In particular, the material should be compatible with cell culture in the long-term if the electrodes are fixed inside the chip. The material must not degrade over time in contact with the cell culture medium. Finally, by choosing a material with a rough surface, the effective surface of the electrodes can be increased, therefore reducing the influence of the double layer capacitance on the measurement. This will allow for more accurate measurement of the TEER, especially for small electrodes since the double layer capacitance is inversely proportional to the electrode area.

## Conclusion

To conclude, TEER measurements and impedance spectroscopy present an easy and non-invasive way to monitor the integrity of biological barriers. Many research groups have made successful attempts at integrating electrodes for these measurements into their OoC designs. This review summarized some of the different electrode and chip designs. Nevertheless, there is a need to further develop OoC with integrated electrodes, particularly ones that mimic the native cellular environment more closely. Additionally, chip designs should be optimized towards up-scalable fabrication and higher throughput. Finally, by standardizing the design, results obtained in different studies can be compared more easily which will lead to a more widespread use of these OoC.

## Author contributions

M. H. wrote the manuscript and L. S. contributed to writing, reviewing, and editing the manuscript.

## Conflicts of interest

There are no conflicts to declare.
